# HIV and HCV Co-Culture Promotes Profibrogenic Gene Expression through an Epimorphin-Mediated ERK Signaling Pathway in Hepatic Stellate Cells

**DOI:** 10.1371/journal.pone.0158386

**Published:** 2016-06-30

**Authors:** Lei Shi, Enqiang Qin, Junnian Zhou, Juanjuan Zhao, Weimin Nie, Tianjun Jiang, Weiwei Chen, Dan Wu, Lei Huang, Liying Liu, Liping Lv, Min Zhao, Zheng Zhang, Fusheng Wang

**Affiliations:** 1 Medical School of Chinese PLA, Beijing, China; 2 Treatment and Research Center for Infectious Diseases, Beijing 302 Hospital, Beijing, China; 3 Beijing Institute of Transfusion Medicine, Beijing, China; 4 Research Center for Clinical and Translational Medicine, Beijing 302 Hospital, Beijing, China; 5 Tumor Radiotherapy Center, Beijing 302 Hospital, Beijing, China; Rosalind Franklin University of Medicine and Science, UNITED STATES

## Abstract

Accelerated fibrosis in patients co-infected with hepatitis C virus (HCV) and human immunodeficiency virus (HIV) has been a major cause of mortality in the highly active anti-retroviral therapy (HAART) era. However, the role of co-infection in accelerating the progression of liver fibrosis, particularly with regard to the effects of co-infection on hepatic stellate cells (HSCs), remains unclear. We hypothesized that HIV and HCV induce liver fibrosis synergistically by altering the regulation of epimorphin production, and thereby indirectly alter HSC function. Here, we examined the effects of epimorphin on HSC proliferation and invasion, and the changes in fibrogenesis-related gene activity in HSCs (LX2) in the presence of inactivated CXCR4-tropic HIV and HCV (JFH1). The combination of HIV and HCV significantly increased epimorphin expression, which increased the proliferation and invasion capabilities of HSCs. Epimorphin also induced the expression of profibrogenic tissue inhibitor of metalloproteinase 1 (TIMP1) in an extracellular signal-regulated kinase (ERK)-dependent manner. These data indicated that the effects of HIV/HCV co-infection on hepatic fibrosis might be mediated in part by EPM. Strategies to limit the expression of EPM might represent a novel therapeutic approach to prevent the progression of hepatic fibrosis during HIV/HCV co-infection.

## Introduction

Hepatitis C virus (HCV) and human immunodeficiency virus (HIV) affect approximately 150 million and 35 million people worldwide, respectively[1, 2]. Given that both viruses are transmitted by similar routes, a large number of people (5–7 million) are co-infected with HIV and HCV[2–4]. Interestingly, HCV does not have a significant effect on the progression of HIV infection; however, HIV accelerates HCV-related liver disease[3, 5, 6]. Despite of the introduction of highly active anti-retroviral therapy (HAART), accelerated liver fibrosis remains a major cause of mortality in HIV and HCV co-infected patients [7]^-^[8]. The most likely mechanisms include direct viral effects; immune/cytokine dysregulation; altered expression levels of matrix metalloproteinase and fibrosis biomarkers; increased oxidative stress and hepatocyte apoptosis; HIV-associated gut depletion of CD4 cells; and microbial translocation[6, 9]. Although HIV does not replicate in human hepatocytes, it does infect CD4 T lymphocytes, macrophages, dendritic cells and hepatic stellate cells (HSCs)[10]. HIV, especially the gp120 protein, can trigger cell signaling pathways in HSCs, immune cell and hepatocytes by interacting with the CXCR4 or CCR5 chemokine receptors[11–13].

Hepatic fibrosis is a wound healing response that occurs in response to liver damage and is the result of an imbalance between the production and dissolution of the extracellular matrix[14, 15]. HSCs are the key contributors to fibrosis[14, 15]. Previous studies suggested that stromal HSCs undergo a phenotypic transformation from a ‘‘quiescent” to an ‘‘activated” state during hepatic fibrosis. This is accompanied by the upregulation of cytoskeletal protein expression (e.g.,α-smooth muscle actin, α-SMA[16]). Activated HSCs secrete a variety of proteins involved in diverse processes, such as signaling transduction, growth factors and soluble mediators, which contribute to accelerated hepatic fibrosis[14, 15]. However, the mechanisms that contribute to liver fibrosis during HIV and HCV co-infection have not been fully explored.

Epimorphin (EPM), also called syntaxin-2, is an extracellular protein that functions as a key epithelial morphoregulator in organs such as the mammary gland, lung, pancreas, intestine and liver[17, 18]. In the liver, EPM is specifically expressed by HSCs, and is involved in liver morphogenesis and regeneration[18–21]. In mice, EPM has been reported to promote chronic inflammation-associated colon carcinogenesis[22]. Our previous work showed that EPM regulates rat liver epithelial stem-like cell differentiation into bile duct cells[23, 24]. More importantly, EPM has the potential to promote hepatocellular carcinoma (HCC) invasion and metastasis by upregulating matrix metalloproteinase (MMP)-9 expression[25]. While EPM is known to be involved in liver development and HCC invasion, its function in hepatic fibrosis during HIV and HCV co-infection has not been characterized.

In the present study, we hypothesized that HIV and HCV cooperatively induce liver fibrosis by altering the production of EPM, which subsequently changes MMP or tissue inhibitors of matrix metalloproteinases (TIMP) expression. To test this hypothesis, we examined the impact of inactivated CXCR4-tropic HIV (NL4-3) and HCV (JFH1) on fibrogenesis-related gene activity in the HSC line LX2. We found that HIV and HCV co-culture upregulated EPM expression, which increased the proliferation and invasion of HSCs. Furthermore, HIV and HCV increased the expression of the profibrogenic gene *TIMP1* by inducing EPM expression through the activation of theERK signaling pathway.

## Materials and Methods

### Cell culture

The human hepatoma cell line, Huh-7.5, was obtained from Dr. C. Rice (Rockefeller University, NY)[26]. Huh-7.5 cells were cultured at 37°C in a humidified atmosphere containing 5% CO_2_ in Dulbecco’s modified Eagle medium (Sigma, St.Louis, Missouri, USA), supplemented with 10% fetal calf serum (Hyclone, UTA, USA), 100 U of penicillin/mL and 100 mg of streptomycin sulfate/mL. The human HSC line, LX-2, was obtained from the Xiang Ya Central Experimental Laboratory of the Central South University (Changsha, China) and cultured in DMEM as described above. To monitor changes in secreted protein levels in the culture supernatant in response to inactivated HIV, cells were cultured in UltraCulture Serum-Free Medium (BioWhittaker, Walkersville, MD, USA) supplemented with 2 mM glutamine (Mediatech, Inc., Herndon, VA, USA).

### HIV stocks

Inactivated HIV supernatant was produced as described previously [13]. HIV-containing (NL4-3, CXCR4-tropic) and mock infected supernatants were heat-inactivated at 56°C for 30 min. The HIV-1 p24 concentration in the viral stock was measured using an Alliance p24 Antigen ELISA kit (PerkinElmer, Waltham, MA, USA). The p24 concentration of the viral stock was 65 ng/mL. The cells were incubated in UltraCulture serum-free medium with or without the supernatant containing HIV (diluted 1:10 in the final volume) for 24 h.

### HCV cell culture infection system

The plasmid pFL-J6/JFH1, encoding the HCV J6/JFH-1 genome, was linearized with *XbaI* for *in vitro* transcription using an Ampliscribe T7 transcription kit (Promega, Madison, WI, USA). The J6/JFH-1 RNA was delivered into Huh-7.5 cells by electroporation (Transfection System Starter Pack, Thermo). The cells were passaged every 3–4 days, and the cells and supernatants were assessed for the presence of HCV. The cell-free virus was propagated in Huh-7.5 cells as described previously[13]. The viral titer in the cell culture supernatant was expressed as focus forming units (ffu) mL^-1^, which was determined by the average number of HCV-NS5A-postitive foci detected at the highest dilutions, as described previously[13]. The HCV positive cell culture supernatant was used to infect naive Huh-7.5 cells at an MOI of 1 for 5–6 h at 37°C in a 5% CO_2_ atmosphere. The cells were then grown in complete DMEM for 5–7 days. In most of the experiments, HCV-infected cells at day 6–7 post infection were used. The cell culture supernatant collected from Huh-7.5 cells expressing replication defective JFH-1/GND was used as a negative control.

### Cellular proliferation assay

Quantification of cellular proliferation was performed using a Cell Counting Assay Kit-8 (CCK-8; Dojindo Molecular Technologies, Gaithersburg, MD, USA), according to the manufacturer's instructions. Briefly, cells were treated with CCK-8 solution (10 μl) and incubated for 1 h. The absorbance was then measured using a spectrophotometer at 490 nm (Bio-Rad). The experiments were performed at least three times and representative data were shown.

### Cell cycle analysis

The cells that were used for DNA content analysis were trypsinized, pelleted by centrifugation (600 × *g* for 5 min), and resuspended in 500 μL of PBS. Ice-cold 70% ethanol (4.5 mL) was slowly added to each cell suspension while gently vortexing to inhibit clumping. Before flow analysis, the cells were repelleted, rinsed with PBS, and incubated for 30 min in a staining buffer containing 0.1% Triton X-100, 0.2 mg/mL RNase A, and 20 μg/mL propidium iodide. The cell cycle phase distribution was determined using a Beckman/Coulter EPICS Elite flow cytometer.

### *In vitro* cell invasion assay

Cells (2 × 10^4^) in 500 μL of MEM medium were added into the upper chamber of a Transwell plate. The lower chamber of the Transwell contained 750 μL of MEM medium supplemented with 15% fetal bovine serum and 10 μg/mL of bovine fibronectin (Invitrogen, Germany). The cells were allowed to migrate through the Transwell membrane for 24 h at 37°C. The membranes were then fixed with 10% neutral-buffered formalin and stained in 10% Giemsa solution. The cells attached to the lower side of the membrane were counted under a high power magnification (200×); ten fields from each membrane were counted. Each experiment was performed in triplicate and three independent experiments were performed.

### Quantitative real-time PCR

Total RNA was extracted using an RNeasy Plus Mini Kit (QIAGEN, Valencia, CA, USA) following the manufacturer's instructions and treated with DNAse I (Promega) for 15 min to remove genomic DNA. CDNA was generated by reverse-transcribing total RNA (1 μg) using oligo (dT) primers and ReverTra Ace reverse transcriptase (Toyobo). Quantitative real-time PCR was performed on an ABI PRISM 7900 system (Applied Biosystems, Foster City, CA, USA) using the SYBR Green Realtime PCR Master Mix plus (Toyobo) to determine the relative levels of gene expression. The expression level of GAPDH was used as the internal control. The primers used in this study are provided in Table 1.

**Table 1:** Primer sequences used for qRT-PCR.

Gene	Forward primer (5’ to 3’)	Reverse primer (5’ to 3’)
*Epimorphin*	CCATCTTCACTTCCGACATTAT	GTGGCATTCATAACATTTCTT
*α-SMA*	AAAAGACAGCTACGTGGGTGA	GCCATGTTCTATCGGGTACTTC
*Col IV*	CAGCCAGACCATTCAGATCC	TGGCGCACTTCTAAACTCCT
*TGF-β*	GCGTGCTAATGGTGGAAAC	GCTGAGGTATCGCCAGGAAT
*MMP-1*	CACAGCTTTCCTCCACTGCTGCT	GGCATGGTCCACATCTGCTCTTG
*MMP-2*	ACCTGGATGCCGTCGTGGAC	TGTGGCAGCACCAGGGCA
*MMP-3*	GTTCCGCCTGTCTCAAGATGA	GGGACAGGTTCCGTGGGTA
*MMP-7*	AAACTCCCGCGTCATAGAAAT	CCCTAGACTGCTACCATCCG
*MMP-9*	TGACAGCGACAAGAAGTG	CAGTGAAGCGGTACATAGG
*MMP-11*	CCGCAACCGACAGAAGAGG	ATCGCTCCATACCTTTAGGGC
*TIMP-1*	AGCAGGGCCTGCACCTGTGTC	TTCAGAGCCTTGGAGCTGGTC
*TIMP-2*	ATGAGATCAAGCAGATAAAGATG	GGTCCTCGATGTCGAGAAACTC
*TIMP-3*	GCTCATCGTGCTCCTGGGCAG	CTCGGTACCAGCTGCAGTAGC
*GAPDH*	GAGTCAACGGATTTGGTCGT	TTGATTTTGGAGGGATCTCG

### Western blotting

Cells were washed twice with PBS and total cellular proteins were extracted using a radioimmunoprecipitation assay (0.5% Nonidet P-40, 10 mmol/L Tris, pH 7.4, 150 mmol/L NaCl, 1% sodium dodecyl sulfate). Whole cell lysates were sonicated, boiled at 95°C for 5 min and then chilled on ice for 10 min. The relative level of protein expression was determined using specific antibodies. Antibodies used for Western blots were: rabbit antibodies against Syntaxin 2 (ab12369, Abcam), α-SMA (Sigma), phospho-FAK (Tyr-397, Biosource International, Camarillo, CA), ERK 1/2, phospho-ERK 1/2 (Thr202/Tyr204), MMP-3 and TIMP-1(Cell Signaling Technology), GAPDH (ab70699, Abcam); and mouse monoclonal antibodies against FAK (Upstate Biotechnology, NY). The immunoreactions were visualized using an enhanced ECL detection kit (Amersham Pharmacia Biotech), exposed to X-ray film and quantified using a video documentation system (Gel Doc 2000, Bio-Rad).

### Construction of lentivirus encoding *EPM* and short hairpin RNA (shRNA) for *EPM*

The human *EPM* cDNA and the shRNA sequences for *EPM* (Forward: TGTTAAAGGCTATTGAACAATTCAAGA, and Reverse: GATTGTTCAATAGCCTTTAACTTTTTTC) were cloned into the lentiviral vector. The respective lentiviral vectors were transfected to 293FT cells for viral packaging. Sixty hours after transfection, the virus was collected to infect target cells to establish stable EPM knockdown and overexpression cell lines.

### Statistical analysis

Data are expressed as the means ± SEM. For multiple comparisons, analysis of variation (ANOVA) or repeated analysis of variation followed by the least squares difference post-hoc test was used. All analyses were performed using SPSS v13.0 (SPSS). A P value < 0.05 was considered statistically significant.

## Results

### HIV and HCV co-culture promoted HSC proliferation and invasion

**Fig 1:**
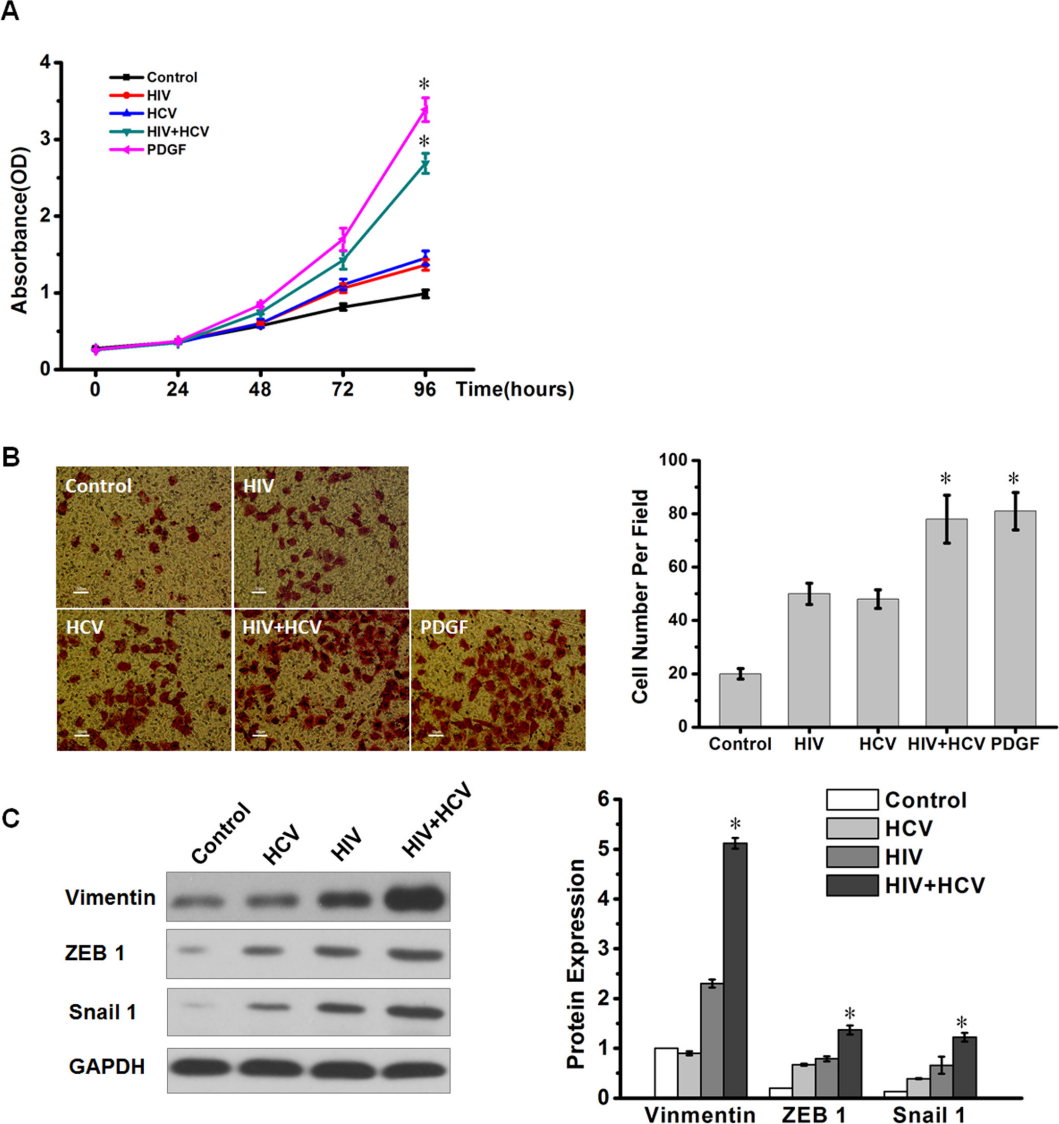
HIV+HCV co-culture promoted HSC proliferation and invasive potential. LX-2 cells were incubated with control medium, HCV (JFH1), inactivated HIV (NL4-3) or HIV and HCV (HIV+HCV). **(A)** Cell proliferation analyzes were performed using a CCK-8 kit *in vitro*. Experiments were performed three times. *P < 0.05 compared with the HIV or HCV group. **(B)** Invasion assays were used to analyze the invasive capability of LX-2 cells. The typical fields in the various groups are shown on the left, and bar graphs are shown on the right. *P < 0.05 compared with the HIV or HCV group. **(C)** Western blotting analysis of the expressions of Vimentin, ZEB1 and Snail 1 in LX-2 cells incubated with control medium, HCV (JFH1), inactivated HIV (NL4-3) or HIV and HCV (HIV+HCV). GAPDH was used as the internal control. *P < 0.05 compared with the HIV or HCV group.

HSCs are the major source of fibrosis following their activation from quiescent vitamin A-rich cells to proliferative and fibrogenic myofibroblasts, including further proliferation and enhanced invasive activity. We used platelet-derived growth factor-BB (PDGF-BB, 20ng/ml) as a positive control for HSC proliferation and invasion. To explore the effects of HIV+HCV on LX-2 cell proliferation, we incubated LX-2 cells with control medium, HCV, HIV, or HIV and HCV (HIV+HCV), respectively for 24, 48, 72 and 96h. The control was a mixture of HCV mock infected and deactivated HIV mock infected supernatants. We performed CCK-8 assays to detect the proliferative ability of HSCs. The growth curves showed that after incubation with HIV+HCV medium, the cell count was significantly increased compared with HIV, HCV or control medium in 72h and 96h (Fig 1A). Compared with the HSCs incubated with control medium alone, the HSCs incubated with HCV had slightly increased percentages of cells in the G2 and M phases of the cell cycle. The percentages of cells in the G2 and M phases of the cell cycle were significantly increased compared with the control in HSCs incubated with HIV+HCV (S1 Fig). Using an *in vitro* cell invasion assay, we investigated the effect of exposure to the viruses on the ability of LX-2 cells to invade. Exposure to HIV+HCV significantly enhanced the invasive potential of LX-2 cells compared with the control, HIV or HCV (Fig 1B). As a positive control, PDGF-BB promoted obvious HSCs proliferation and invasion. We also detected some invasion markers (Vimentin, ZEB1 and Snail1) and found that HIV+HCV co-culture increased the protein abundance of these markers (Fig 1C). These results suggested that HIV+HCV co-culture promoted HSC proliferation and invasion to a significant extent.

### HIV and HCV co-culture increased mRNA and protein expression of alpha smooth muscle actin (α-SMA), collagen IV, transforming growth factor beta (TGF-β) and EPM in HSCs

**Fig 2:**
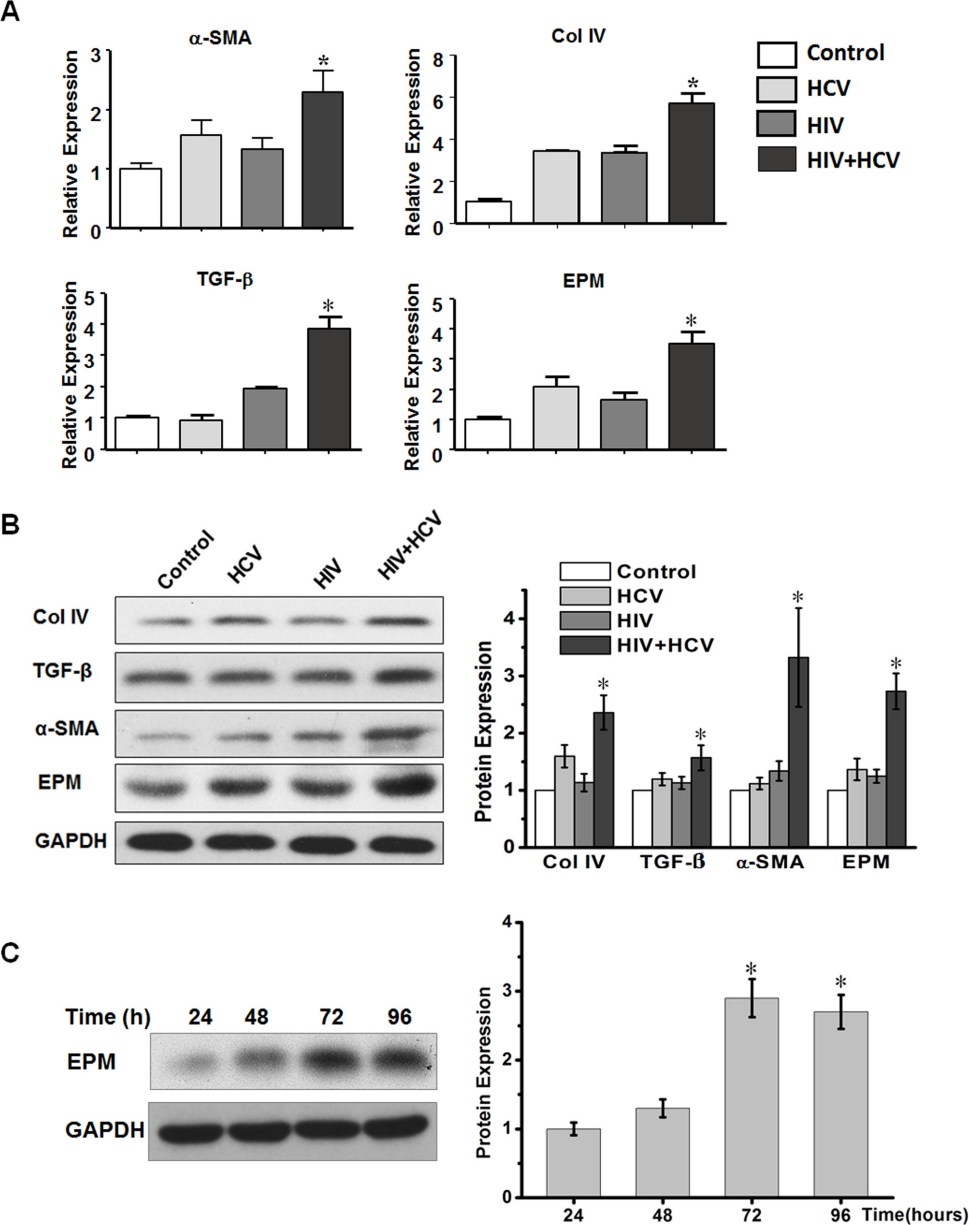
HIV+HCV co-culture increased mRNA and protein expression levels of α-SMA, collagen IV, TGF-β, and EPM in HSCs. **(A)** QRT-PCR analysis of fibrogenesis-related genes in LX-2 cells. MRNA expression levels of *α-SMA*, *collagen IV*, *TGF-β*, *and EPM* were significantly upregulated after culture with HIV+HCV. *GAPDH* was used as the internal control. *P < 0.05 compared with the HIV or HCV group. **(B)** Western blotting analysis of the fibrogenesis-related protein expression levels in LX-2 cells. EPM, α-SMA, collagen IV, and TGF-β levels were upregulated after 72 h of co-culture with HIV+HCV. GAPDH was used as an internal control. **(C)** Western blotting analysis of EPM protein expression levels in LX-2 cells incubated with HIV+HCV at the indicated time points (h). GAPDH was used as an internal control. Bar graphs are shown on the right. * P < 0.05 compared with 24h.

TGF-β is a central mediator of liver fibrogenesis[27, 28]. In patients, both HCV mono-infection and HIV+HCV co-infection are associated with significantly increased expression of TGF-β in the liver and serum[6]. To explore the effects of HIV+HCV co-culture on the mRNA and protein expression levels of collagen, TGF-β, α-SMA, collagen IV, and EPM, we performed qRT-PCR and western blotting after 72 h of exposure to the viruses. The expression levels of α-SMA, TGF-β, and collagen IV were modestly increased after culture with HIV or HCV separately. Interestingly, EPM, an HSC-specific gene product, accumulated to a high level after co-culture with HIV+HCV. The EPM mRNA expression levels were increased by approximately 3.8-fold (p < 0.01) compared with untreated HSC cells (Fig 2A) and the protein levels showed a similar trend (Fig 2B). We performed western blotting to further confirm EPM expression at different times after co-culture with HIV+HCV. The results showed that EPM was upregulated with time (Fig 2C). Compared with 72h, the expression level was slightly decreased at 96h, which might be caused by cell status. Taken together, these data demonstrated that HIV+HCV co-culture increased EPM expression in activated HSCs.

### HIV and HCV co-culture promoted HSC proliferation and invasion in an EPM dependent manner *in vitro*

**Fig 3:**
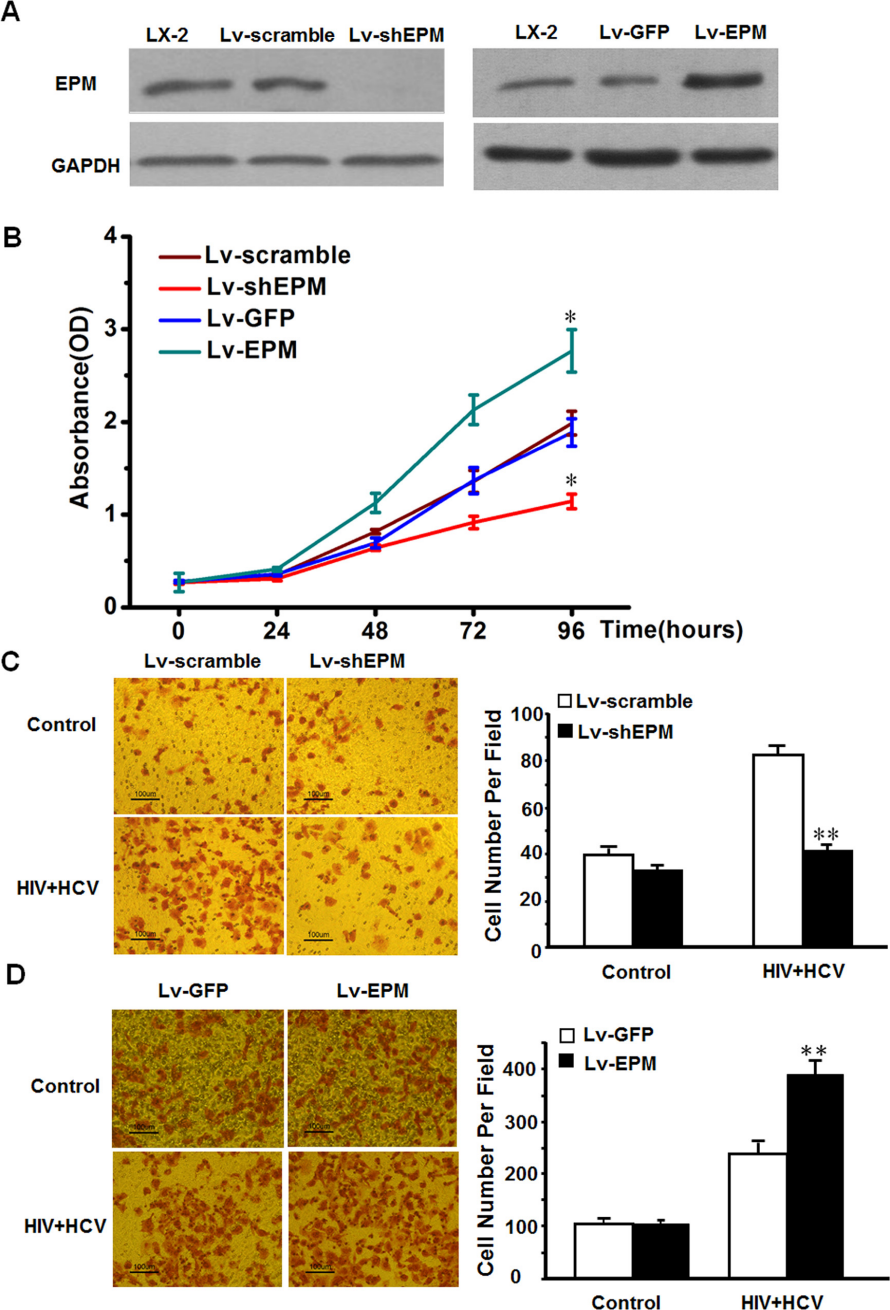
HIV+HCV co-culture promoted HSC proliferation and invasion in an EPM-dependent manner *in vitro*. **(A)** Western blotting analysis of EPM protein expression in LX-2 cells with EPM knockdown (Lv-shEPM) and EPM overexpression (Lv-EPM). The empty vector-transfected LX-2 cells were used as controls. **(B)** Cell proliferation analyses were performed using a CCK-8 kit in LX-2 cells with EPM knockdown (Lv-shEPM) and EPM overexpression (Lv-EPM). The empty vector-transfected LX-2 cells were used as controls. *P < 0.05 compared with the HIV or HCV group. **(C)** *In vitro* cell invasion assay of LX-2 cells with EPM knockdown after HIV+HCV co-culture. The empty vector-transfected LX-2 cells were used as controls. **P < 0.01 compared with Lv-scramble. **(D)** *In vitro* cell invasion assay of LX-2 cells with EPM overexpression after HIV+HCV co-culture. The empty vector-transfected LX-2 cells were used as controls. **P < 0.01 compared with Lv-GFP. Experiments were performed three times.

To assess whether EPM was responsible for the pro-fibrotic effects of HIVHCV co-culture, we generated HSCs with stable EPM knockdown or overexpression, as described previously[25](Fig 3A). When EPM was depleted by lentivirus shRNA, the HSCs activation-related genes such as TGF-β, α-SMA, and collagen IV were significantly repressed (S2 Fig). We then performed CCK-8 assays, cell cycle assays and cell invasion assays to determine whether EPM affected cell proliferation and invasion. EPM knockdown significantly reversed the HIV+HCV-mediated increase in cell proliferation, while overexpression of EPM further increased the HIV+HCV-mediated proliferation in LX-2 cells (Fig 3B and S3 Fig). Similarly, EPM knockdown reduced the ability of LX-2 cells to invade through a Transwell following exposure to HIV+HCV (Fig 3C), while overexpression of EPM increased the invasiveness of LX-2 cells exposed to HIV+HCV (Fig 3D). These results suggested that EPM is involved in the pro-fibrotic effects of HIVHCV co-culture in LX-2 cells.

### HIV and HCV co-culture increased TIMP-1 mRNA and protein expression via an EPM-dependent signaling pathway

**Fig 4:**
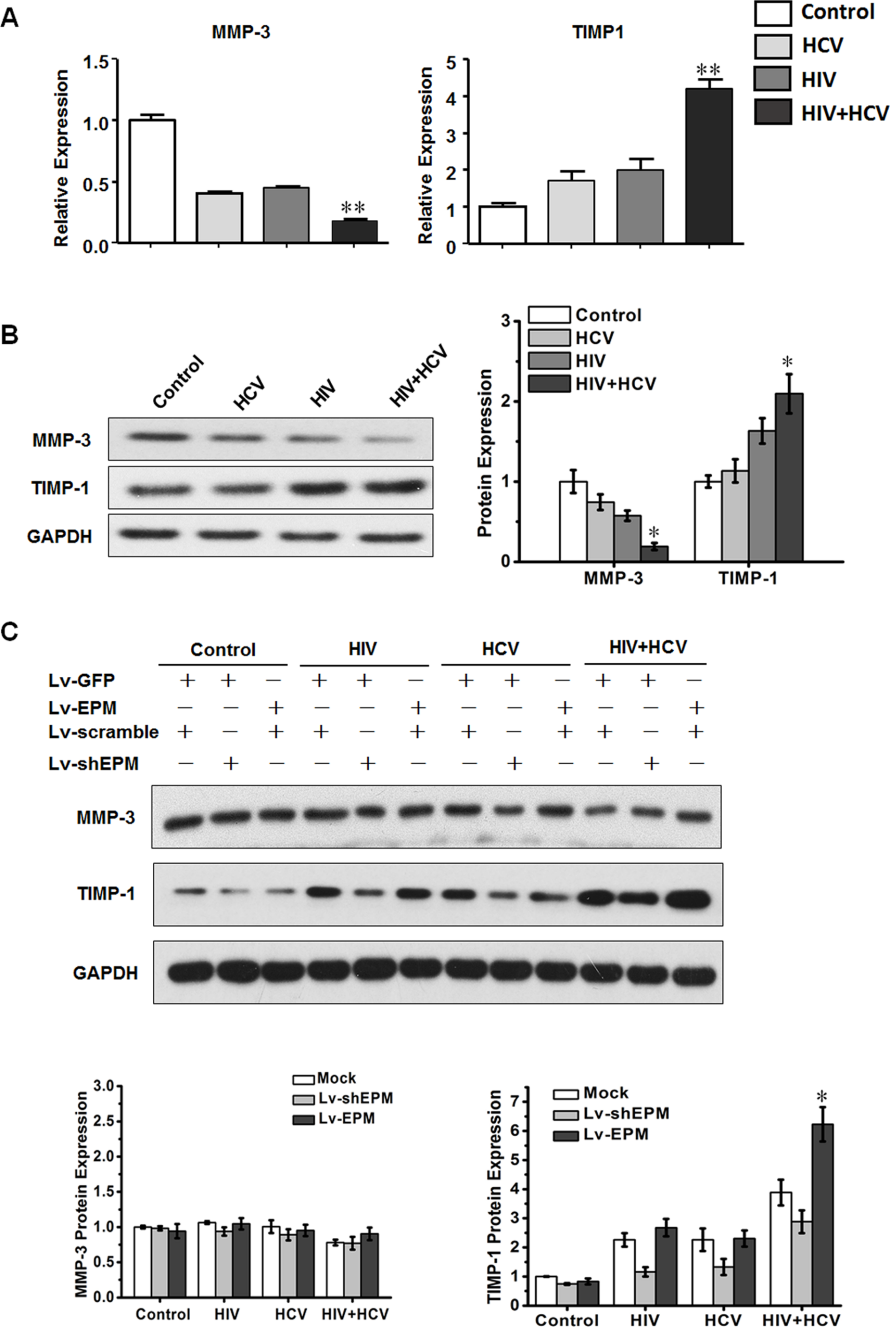
HIV+HCV co-culture increased tissue inhibitors of MMP 1 (TIMP-1) expression levels via an EPM dependent signaling pathway. **(A)** QRT-PCR analysis of *TIMP-1* and *MMP-3* expression levels in LX-2 cells incubated with control medium, HCV (JFH1), inactivated HIV (NL4-3) or HIV and HCV (HIV+HCV). *GAPDH* was used as the internal control. **P < 0.01 compared with the HIV or HCV group. **(B)** Western blotting analysis of the expressions of TIMP-1 and MMP-3 in LX-2 cells incubated with control medium, HCV (JFH1), inactivated HIV (NL4-3), or HIV and HCV (HIV+HCV). Bar graphs are shown on the right. *P < 0.05 compared with the HIV or HCV group. **(C)** Western blotting analysis of the expressions of TIMP-1 and MMP-3 in LX-2 cells, which were transfected with EPM knockdown (Lv-shEPM, with Lv-scramble as the control) and EPM overexpression (Lv-EPM, with Lv-GFP as the control), incubated with control medium, HCV (JFH1), inactivated HIV (NL4-3) or HIV and HCV (HIV+HCV). Bar graphs are shown on the right. Mock: Lv-scramble+ Lv-GFP. *P < 0.05 compared with mock or Lv-shEPM in the HIV+HCV group.

To determine whether HIV+HCV co-infection affected liver fibrosis in other ways, we further examined the effects of HIV+HCV co-culture on MMP and TIMP expression. HSCs incubated with HCV or HIV alone exhibited minimal increases in *TIMP-1* mRNA and protein expression compared with medium-treated HSCs. However, HSCs incubated with HIV+HCV showed significantly increased levels of *TIMP-1* mRNA (Fig 4A) and protein (Fig 4B) expression compared with medium-treated HSCs. MMP-3 was also dramatically decreased in HSCs treated with HIV+HCV. Compared with the HIV or HCV group, the expressions of MMP-1, MMP-2, MMP-7, MMP-9, MMP-11, TIMP-2 and TIMP-3 in the HIV+HCV group were not statistically different (S4 Fig).

To determine whether EPM facilitated the expression of genes related to hepatic fibrosis, the protein levels of MMPs and TIMPs were determined in LX-2 cells stably overexpressing EPM or LX-2 cells with EPM knockdown. Only TIMP-1 was dramatically upregulated by EPM (Fig 4C); expression of MMP-3 was not significantly altered after EPM transfection (Fig 4C). These results suggested that EPM could promote hepatic fibrosis by regulating TIMP-1 expression. Given the important role of TIMP-1 in the process of fibrogenesis, we hypothesized that HIV+HCV co-infection produces an environment that accelerates liver fibrosis in an EPM-dependent manner in the cell model.

### The ERK signaling pathway is involved in EPM-mediated TIMP-1 expression

**Fig 5:**
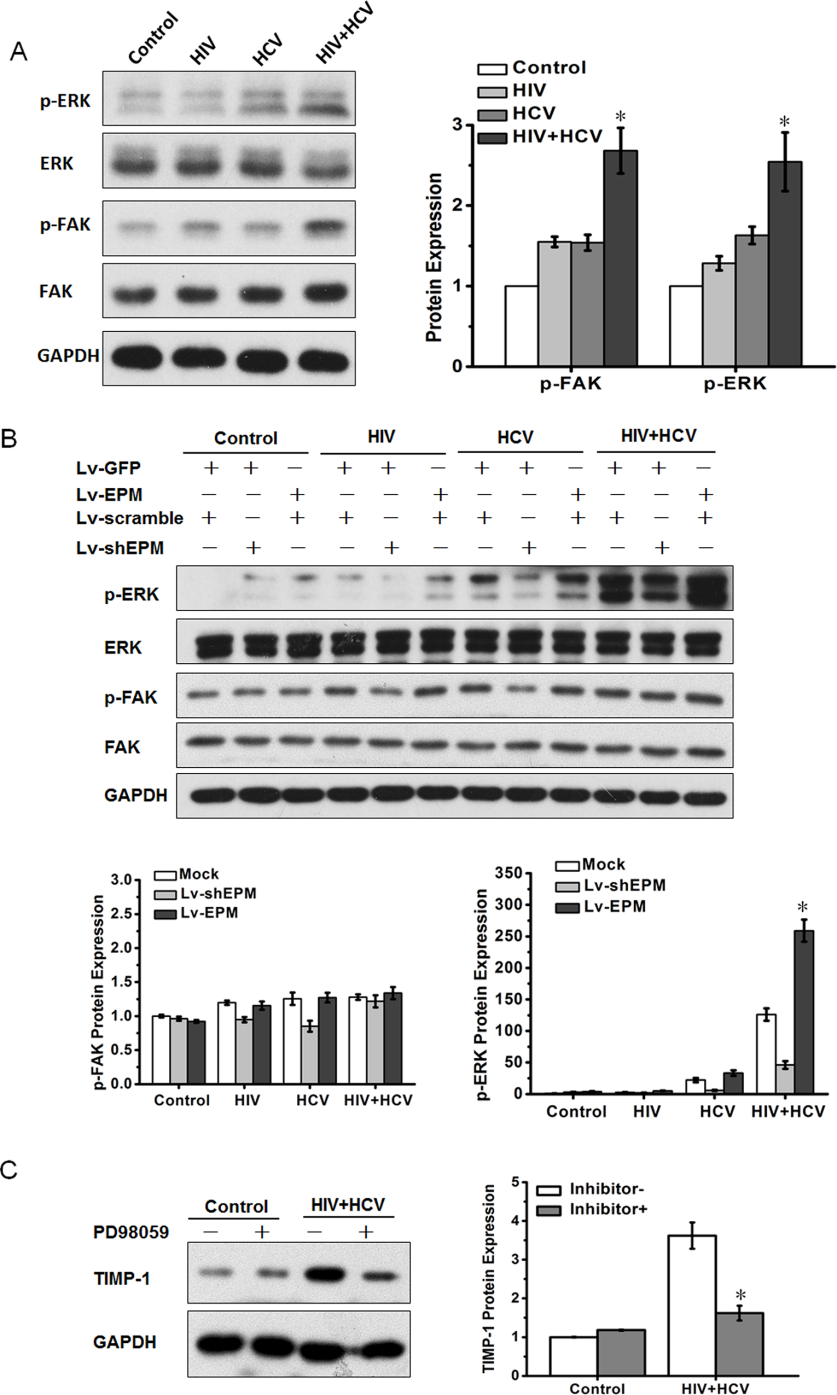
EPM induced TIMP-1 expression via ERK activation. **(A)** Western blotting analysis of total protein and phosphorylation levels of FAK and ERK1/2 in LX-2 cells after culture with control medium, HCV, HIV or HIV+HCV, respectively. Bar graphs are shown on the right. **(B)** Western blotting analysis of total protein and phosphorylation levels of FAK and ERK1/2 in LX-2 cells, which were transfected with EPM knockdown (Lv-shEPM, with Lv-scramble as the control) and EPM overexpression (Lv-EPM, with Lv-GFP as the control), incubated with control medium, HCV (JFH1), inactivated HIV (NL4-3) or HIV and HCV (HIV+HCV). Bar graphs are shown on the right. **(C)** LX-2 cells were treated with the ERK inhibitor, PD98059 (50 μM) for 24 h, then TIMP-1 expression was assessed using western blotting. Bar graphs are shown on the right.

It has been reported that EPM binds to α-integrin-containing receptors, leading to activation of the FAK-ERK signaling pathway and induction of epithelial morphogenesis[29]. Our previous studies confirmed that FAK and ERK phosphorylation are key factors in EPM-induced bile duct formation from liver epithelial stem-like cells[24] and in EPM-induced metastasis of HCC[25]. Therefore, we hypothesized that EPM might regulate TIMP-1 expression in HIV+HCV co-culture through FAK or ERK signaling. The phosphorylation levels of FAK and ERK1/2 were modestly increased after exposure to HIV or HCV, respectively; however, HIV+HCV co-culture significantly increased the phosphorylation levels of both FAK and ERK1/2 (Fig 5A). The ERK signaling pathway was further activated in EPM-transfected LX-2 cells, and was inhibited in EPM-knockdown LX-2 cells (Fig 5B). Meanwhile, there was no significant change in the expression level of FAK. The ERK inhibitor PD98059 (50 μM) attenuated the activation of the ERK pathway, and inhibited TIMP1 expression in LX-2 cells (Fig 5C). These data indicated that EPM regulates TIMP1 expression in an ERK signaling pathway-dependent manner.

## Discussion

Compared with mono-infected HCV patients, there are more liver events, such as liver fibrosis/cirrhosis, liver failure and liver-related death, in HIV+HCV co-infected patients[6]. Despite some direct and indirect mechanisms could explain the accelerated liver fibrosis in HIV+HCV co-infected patients[8, 9], the profibrogenic mechanisms of HIV+HCV co-infection have not been fully explored.

Among the cells with contributions to liver fibrosis, activated HSCs are the key fibrogenic effectors in the liver[30]. HSCs account for 5–8% of the total cells in a healthy liver and play important roles in liver development, differentiation and regeneration[31, 32]. Progression of liver fibrosis has been attributed to HSC proliferation and invasion[31]. Increased matrix production by activated HSCs is also the most direct way of causing hepatic fibrosis[15, 32, 33].

The HIV envelope protein gp120 has been shown to trigger cell signaling pathways in the liver through interactions with CCR5 and CXCR4 expressed on the surface of HSCs[11, 12]. HIV can infect HSCs directly, leading to increased collagen, TGF-β, and monocyte chemoattractant protein 1 (MCP-1) production, although the mechanism by which HIV gains entry to these cells is unclear, because this effect is not blocked by antibodies against CD4, CXCR4, or CCR5[10, 13].

In the present study, we found that HIV+HCV co-culture increased HSC proliferation and invasion, induced the expression of several genes (such as collagen IV and TGF-β) and increased the levels of EPM. We also confirmed that the expression of MMP-3 was reduced and TIMP-1 was increased by HIV+HCV co-culture. We found that upregulation of TIMP-1 via ERK activation was required for EPM-mediated liver fibrosis, because blockade of EPM using an shRNA in HSCs significantly reduced ERK activation and TIMP-1 expression. However, MMP3 was not regulated by EPM. These data suggested that the increased level of EPM in activated HSCs might contribute to hepatic fibrosis. Together with a study from Lin et al., in which they report that HIV and HCV independently affected the progression of hepatic fibrosis through the ROS-NF-κB-TIMP1 pathway[34], our data showed that HIV+HCV co-culture directly affected HSCs in the liver, leading to increased production of profibrogenic cytokines and ECM. These factors might contribute to the accelerated liver fibrosis observed in HIV+HCV co-infected patients[11, 34].

In contrast to our findings, Pritchett et al. reported that EPM was decreased during HSC activation[35]. One possibility is that the rat liver injury model triggered by carbon tetrachloride that Pitchett used was different from the human viral infection model used here. Another possibility is that EPM is differentially expressed in the various phases of HSC activation. For example, EPM expression was transiently decreased in the early-phase and strikingly enhanced in the recovery phase[36]. Our current findings are consistent with our previous observation that EPM was upregulated in activated HSCs[25].

Our study has several limitations. We were unable to measure the expression of related genes in the human tissues because of a lack of liver biopsy tissue. In addition, we have not addressed why EPM was significantly increased by HIV+HCV co-culture. The complex nature of the immune system might result in the secretion some biofactors that enhance the expression of EPM in HSCs; however, the phenotype and mechanisms still need to be verified in patients co-infected with HIV and HCV. Future studies are warranted to address these questions. Despite these limitations, we have demonstrated that HIV and HCV mono-infection and co-infection increase the level of EPM, which might induce the expression of profibrogenic genes, such as TIMP1, via the ERK signaling pathway and subsequently increase the proliferation and invasion of HSCs. These findings support a role of EPM as an important biomarker in activated HSCs during hepatic fibrosis with HIV+HCV co-infection. In addition, interrupting the EPM-ERK-TIMP1 pathway might be a useful therapeutic approach to control hepatic fibrosis induced by HIV+HCV co-infection.

## Supporting Information

S1 Fig Cell cycle analysis of HSC cells after HIV+HCV co-culture. LX-2 cells were incubated with control medium, HCV (JFH1), inactivated HIV (NL4-3) or HIV and HCV (HIV+HCV). After HIV+HCV co-culture, the percentage of the cells in the G2/M phase was significantly increased compared with that of the HIV or HCV group, while the percentage of the cells in the S phase was significantly decreased compared with that of the HIV or HCV group. **P < 0.01 compared with the HIV or HCV group.
(TIF)

S2 Fig QRT-PCR analysis of *TGF-β*, *α-SMA* and *Col IV* expression levels in LX-2 cells with EPM knockdown (Lv-shEPM) after HIV+HCV co-culture. *TGFβ*, *αSMA*, and *Col IV* were significantly repressed when EPM was depleted by lentivirus shRNA. *GAPDH* was used as the internal control in qRT-PCR. **P < 0.01 compared with Lv-scramble in the control group. ##P < 0.01 compared with Lv-scramble in the HIV+HCV group.
(TIF)

S3 Fig Cell cycle analysis of LX-2 cells with EPM knockdown and EPM overexpression after HIV+HCV co-culture. **(A)** Cell cycle analysis of LX-2 cells (Lv-scramble and Lv-shEPM) after control medium culture or HIV+HCV co-culture. EPM knockdown significantly reversed the HIV+HCV-mediated proliferation of LX-2 cells. *P < 0.05 compared with Lv-scramble in the HIV+HCV group. **(B)** Cell cycle analysis of LX-2 cells (Lv-GFP and Lv-EPM) after HIV+HCV co-culture. Overexpression of EPM further increased the proliferation of LX-2 cells compared with HIV+HCV. *P < 0.05 compared with Lv-GFP in the HIV+HCV group.
(TIF)

S4 Fig QRT-PCR analysis of *MMP* and *TIMP* expression levels. LX-2 cells were incubated with control medium, HCV (JFH1), inactivated HIV (NL4-3) or HIV and HCV (HIV+HCV). Compared with the HIV or HCV group, the expression of MMP-1, MMP-2, MMP-7, MMP-9, MMP-11, TIMP-2 and TIMP-3 in the HIV+HCV group were not statistically different. *GAPDH* was used as the internal control for qRT-PCR.
(TIF)
