# Supplementary figures and images for: HIV and HCV Co-Culture Promotes Profibrogenic Gene Expression through an Epimorphin-Mediated ERK Signaling Pathway in Hepatic Stellate Cells
Source: PLoS One. 2016 Jun 30;11(6):e0158386. doi: 10.1371/journal.pone.0158386 (PMC4928874; doi:10.1371/journal.pone.0158386)

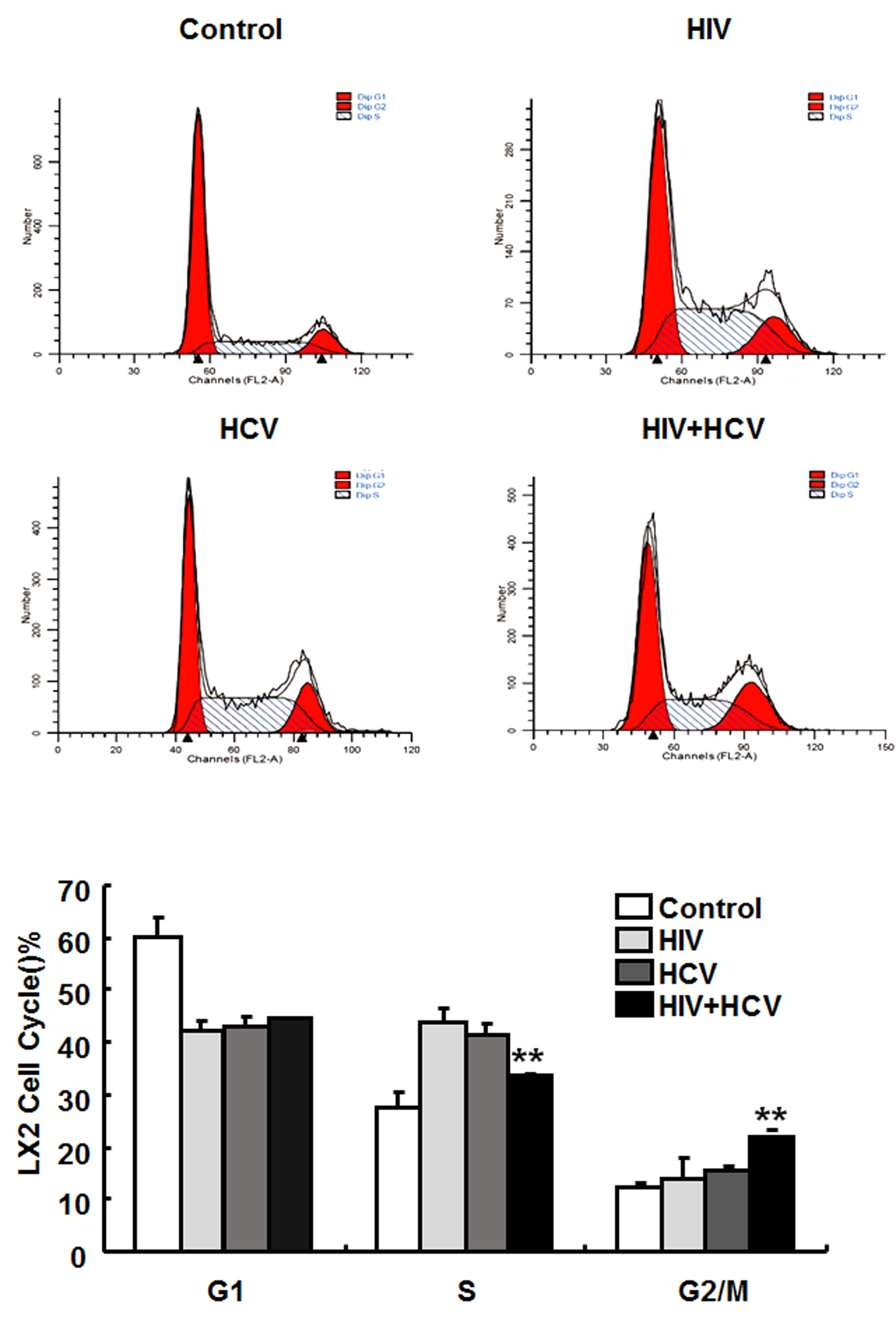

Supplement: S1 Fig — LX-2 cells were incubated with control medium, HCV (JFH1), inactivated HIV (NL4-3) or HIV and HCV (HIV+HCV). After HIV+HCV co-culture, the percentage of the cells in the G2/M phase was significantly increased compared with that of the HIV or HCV group, while the percentage of the cells in the S phase was significantly decreased compared with that of the HIV or HCV group. **P < 0.01 compared with the HIV or HCV group. (TIF) [file pone.0158386.s001.tif]

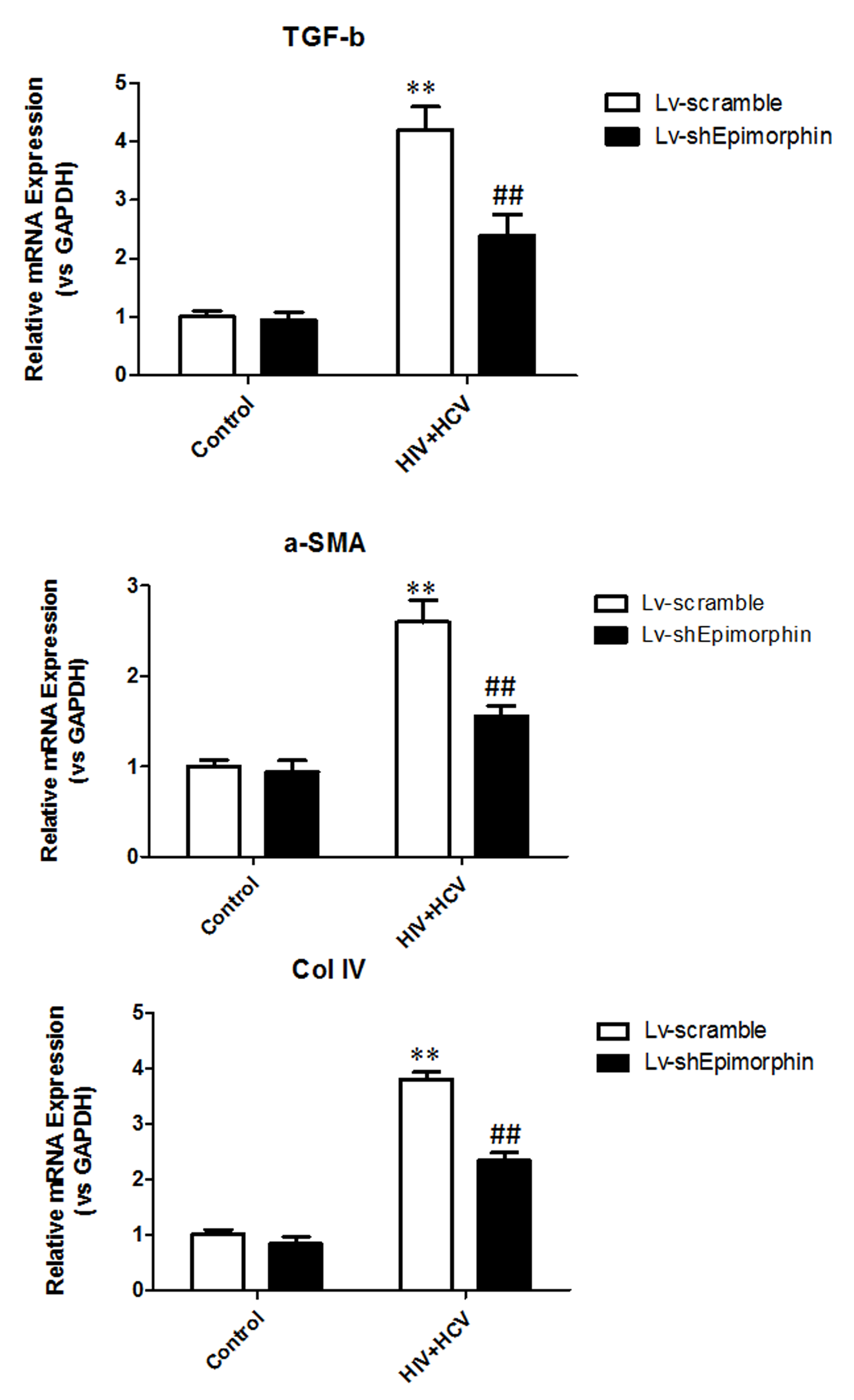

Supplement: S2 Fig — TGFβ, αSMA, and Col IV were significantly repressed when EPM was depleted by lentivirus shRNA. GAPDH was used as the internal control in qRT-PCR. **P < 0.01 compared with Lv-scramble in the control group. ##P < 0.01 compared with Lv-scramble in the HIV+HCV group. (TIF) [file pone.0158386.s002.tif]

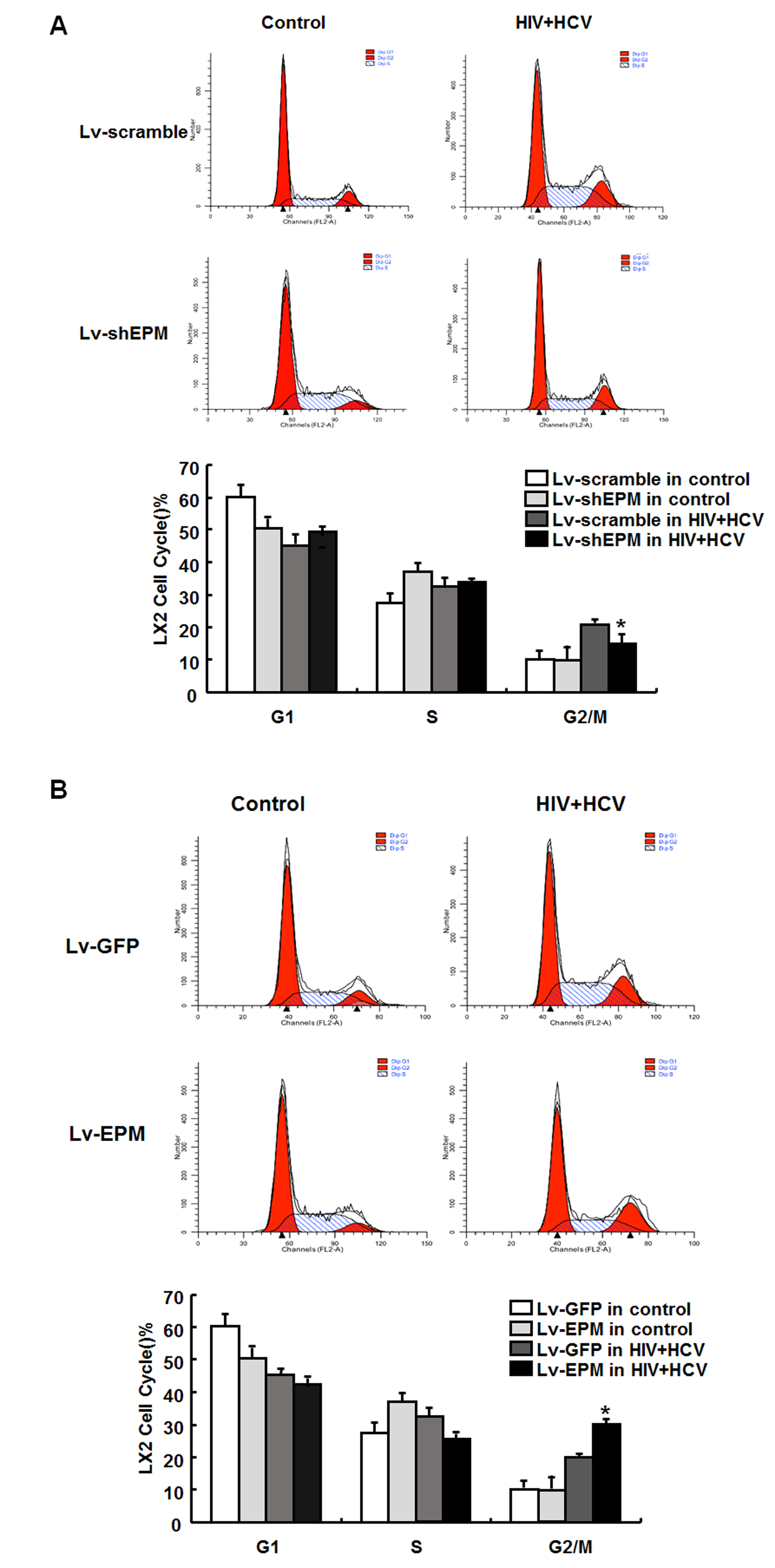

Supplement: S3 Fig — (A) Cell cycle analysis of LX-2 cells (Lv-scramble and Lv-shEPM) after control medium culture or HIV+HCV co-culture. EPM knockdown significantly reversed the HIV+HCV-mediated proliferation of LX-2 cells. *P < 0.05 compared with Lv-scramble in the HIV+HCV group. (B) Cell cycle analysis of LX-2 cells (Lv-GFP and Lv-EPM) after HIV+HCV co-culture. Overexpression of EPM further increased the proliferation of LX-2 cells compared with HIV+HCV. *P < 0.05 compared with Lv-GFP in the HIV+HCV group. (TIF) [file pone.0158386.s003.tif]

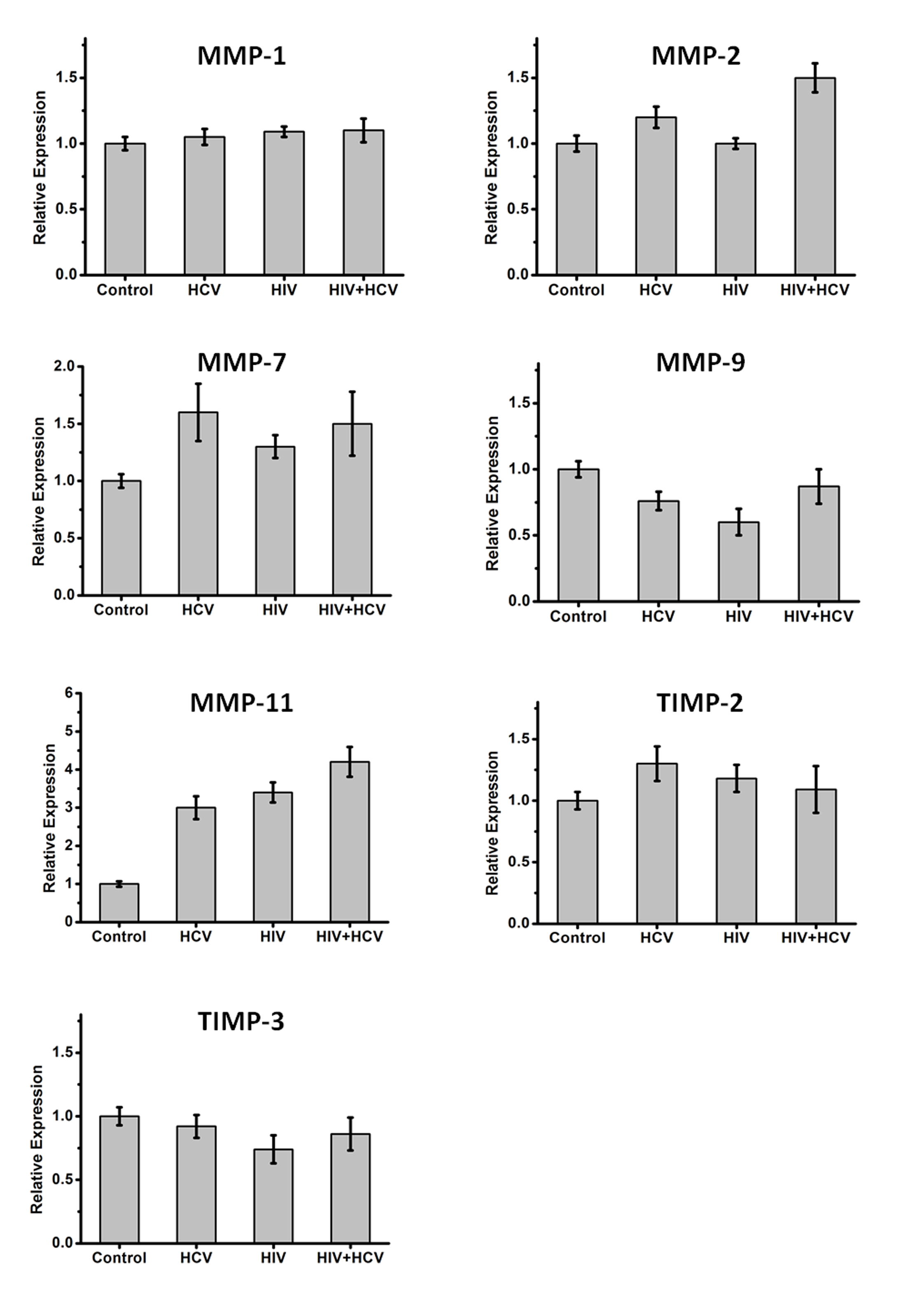

Supplement: S4 Fig — LX-2 cells were incubated with control medium, HCV (JFH1), inactivated HIV (NL4-3) or HIV and HCV (HIV+HCV). Compared with the HIV or HCV group, the expression of MMP-1, MMP-2, MMP-7, MMP-9, MMP-11, TIMP-2 and TIMP-3 in the HIV+HCV group were not statistically different. GAPDH was used as the internal control for qRT-PCR. (TIF) [file pone.0158386.s004.tif]
